# Left Atrial Appendage Exclusion for Stroke Prevention in Atrial Fibrillation

**DOI:** 10.1155/2012/610827

**Published:** 2012-10-16

**Authors:** Taral K. Patel, Clyde W. Yancy, Bradley P. Knight

**Affiliations:** Division of Cardiology, Department of Internal Medicine, Feinberg School of Medicine, Northwestern University, 251 East Huron Street, Feinberg 8-503E, Chicago, IL 60611, USA

## Abstract

The public health burden of atrial fibrillation (AF) and associated thromboembolic stroke continues to grow at alarming rates. AF leads to a fivefold increase in the risk of stroke. Therefore, stroke prevention remains the most critical aspect of AF management. Current standard of care focuses on oral systemic anticoagulation, most commonly with warfarin and now with newer agents such as dabigatran, rivaroxaban, and apixaban. However, the challenges and limitations of oral anticoagulation have been well documented. Given the critical role of the left atrial appendage (LAA) in the genesis of AF-related thromboembolism, recent efforts have targeted removal or occlusion of the LAA as an alternative strategy for stroke prevention, particularly in patients deemed unsuitable for oral anticoagulation. This paper highlights recent advances in mechanical exclusion of the LAA. The problem of AF and stroke is briefly summarized, followed by an explanation for the rationale behind LAA exclusion for stroke prevention. After briefly reviewing the history of LAA exclusion, we highlight the most promising LAA exclusion devices currently available. Finally, we discuss future challenges and opportunities in this growing field.

## 1. Introduction: Atrial Fibrillation and Stroke

 Atrial fibrillation (AF) is the most common arrhythmia in modern clinical practice, currently affecting up to 5 million people in the United States [1, 2]. The prevalence rises sharply with age, from approximately 1% among people aged 55–59 years to over 10% among those aged greater than 80 years [3]. Importantly, the burden of AF is expected to rise threefold by 2050 to an estimated 12–16 million Americans [4].

 The most feared clinical consequence of AF is stroke due to thromboembolism. Stroke is the third leading cause of death and the number one cause of major disability in the United States [5]. AF is a powerful risk factor for stroke; a diagnosis of AF increases stroke risk fivefold and conveys an overall stroke rate of 5% per year [5]. Of the estimated 800,000 annual strokes in the USA, the percentage attributable to AF ranges from 1.5% (50–59 years old) to 23.5% (80–89 years old) [5]. As AF is commonly silent and undiagnosed, the influence of AF on stroke is almost certainly underestimated.

## 2. The Role of the Left Atrial Appendage

 AF promotes thromboembolism through a variety of mechanisms, most significantly mechanical dysfunction in the atria leading to impaired blood flow and stasis. Additional factors including endothelial dysfunction, inflammation, platelet activation, and a hypercoagulable state have also been implicated [6–8]. The left atrial appendage (LAA) is particularly vulnerable to thrombus formation due to its complex anatomy and low blood flow during AF. A review of 23 studies found that thrombi were present in 17% of patients with nonrheumatic AF, of which 91% were located in the LAA [9]. A consistent theme has emerged that the vast majority of strokes due to AF represent thromboembolism originating from the left atrial appendage.

Johnson et al. described the LAA as “our most lethal human attachment” [10]. The LAA is derived from the left atrium and forms a blind pouch approximately 2–4 cm long. Most commonly, it lies on the anterior surface of the heart although variation is present. The neck of the LAA is relatively narrow and the endocardial surface is irregular due to pectinate muscles. The number of lobes can vary; one autopsy survey of 500 patients showed that 77% of LAA had two or three lobes while 20% had one lobe [11]. This trabeculated and crypt-rich structure provides an ideal substrate for stasis and clotting. Magnetic resonance imaging and transesophageal echocardiography (TEE) have shed light on the influence of LAA anatomy on the risk of thrombus formation. There are signals that larger LAA ostia (perhaps due to a lower emptying velocity), larger neck diameter, and greater length all portend a higher risk of stroke [12].

## 3. Stroke Prevention: Oral Anticoagulation and Its Limitations

Stroke prophylaxis is one of the pillars of AF management. The current standard of care for stroke prevention in AF is oral systemic anticoagulation [13]. Importantly, stroke risk is not homogenous across the entire population of AF patients. Therefore, the decision to anticoagulate is based on a patient-by-patient assessment of stroke risk in the presence of known clinical risk factors. The most widely used risk assessment tool for nonvalvular AF is the CHADS_2_ scoring system, which incorporates the risk factors of congestive heart failure, hypertension, age over 75 years, diabetes, and prior stroke or transient ischemic attack [14]. Aspirin therapy is recommended for a CHADS_2_ score of 0, systemic anticoagulation is recommended for a CHADS_2_ score of 2 or higher, and either option is reasonable for a CHADS_2_ score of 1. This strategy attempts to balance the bleeding risk from systemic anticoagulation with the thromboembolic stroke risk from untreated AF across the spectrum of CHADS_2_ scores. The newer CHADS_2_-VASc score, which adds the risk factors of vascular disease and female gender, has become useful to further refine stroke risk in patients with an otherwise low CHADS_2_ score [15]. Oral anticoagulation has been undeniably effective as a stroke reduction strategy. Warfarin, the predominant anticoagulant in clinical use, was shown to reduce AF-related stroke by 64% in a large meta-analysis [16].

 However, the widespread use of systemic anticoagulation in AF has brought to light important limitations and disadvantages of this management strategy. Most notably, systemic anticoagulation has the unavoidable consequence of elevated bleeding risk. This has led to relative or absolute contraindications to anticoagulation in up to 40% of AF patients, usually due to a history of significant bleeding or a perceived elevated risk of falls and trauma [17, 18]. Aside from bleeding risk, anticoagulation use is further limited by the inconvenience of frequent blood testing and widespread interactions with food and other medications. In fact, anticoagulation is not utilized in up to 50% of eligible AF patients, often due to the limitations highlighted [19]. Furthermore, patients who are actually treated with warfarin spend up to half of the treatment time outside the therapeutic range [20]. This means that the full potential of warfarin to reduce stroke risk has never been fully realized.

Largely in response to the challenges of using warfarin, the newer oral anticoagulants dabigatran (a direct thrombin inhibitor) and rivaroxaban (a factor Xa inhibitor) have been recently developed and are now commercially available. A third agent, apixaban, has also been evaluated in a large clinical trial but has yet to gain the approval of the Food and Drug Administration (FDA) [21]. These newer agents minimize food and drug interactions and eliminate the need for INR monitoring, thus increasing the ease of administration and compliance. Unfortunately, they still suffer from comparable bleeding risk and a not insignificant level of drug intolerance; the two-year discontinuation rates for dabigatran and rivaroxaban are 21% and 24%, respectively [22, 23]. In addition, unlike warfarin, the newer agents are not easily reversible with blood product transfusion, raising serious concerns in the event of a bleeding incident. Finally, the novel agents come at a significantly higher cost than warfarin. Whether these agents are truly cost-effective in comparison to warfarin is an open question.

## 4. Stroke Prevention: Targeting the Left Atrial Appendage

 While oral anticoagulation options have certainly improved, there remains another glaring question: is systemic anticoagulation the best strategy for treating a pathological process that is largely focal, namely, thromboembolism originating from the LAA? Theoretically, a strategy focused on excluding the LAA should offer similar stroke prophylaxis while also eliminating the disadvantages of systemic anticoagulation, not the least of which is the lifelong commitment to daily medication. LAA exclusion would be an especially appealing option for patients with intolerance or contraindications to anticoagulation. Therefore, in recent years interest has emerged in mechanical exclusion of the LAA as an alternative strategy for AF stroke prevention. 

## 5. LAA Exclusion: Surgical Techniques

 Left atrial appendage exclusion is not a new idea. The first report of LAA exclusion in the surgical literature was in 1949, when Madden published his account of LAA removal in two patients as a prophylactic measure against recurrent arterial emboli [24]. The high complication rate of the procedure prevented its widespread adoption until the 1990s, when interest in the procedure was rekindled by the development of the Cox-Maze III procedure, which included removal of the LAA [25]. Surgical techniques have evolved around the two basic strategies of LAA exclusion (using various suture techniques) and LAA excision (via surgical stapler or removal with oversewing).

 The data for surgical LAA exclusion consist primarily of retrospective case series and case reports. An important problem in the interpretation of these data is that surgical techniques are nonuniform across the literature. Outcomes measurements, especially pertaining to visual confirmation of surgical success, are also variable. The use of transesophageal echocardiography, considered the gold standard for LAA visualization, is absent in many reports. A large review found that surgical success is highly technique and operator dependent, with complete closure rates ranging from 17% to 93% [26]. Excision and oversewing appears to demonstrate the best results. Of note, no data exist to support the notion that surgical LAA exclusion prevents future strokes; that question is the subject of an ongoing clinical trial (LAAOSII; http://clinicaltrials.gov/ NCT00908700). 

The high morbidity of surgical LAA exclusion has prevented its adoption as a stand-alone procedure. Thus, current guidelines limit surgical LAA exclusion as an adjunctive procedure during mitral valve or Maze surgery [27].

## 6. LAA Exclusion: Percutaneous Techniques

 A minimally invasive percutaneous approach to LAA exclusion should theoretically reduce the procedural risks inherent in open surgical techniques. Over the last decade, several percutaneous LAA exclusion devices have been developed and tested in humans. The procedures involve transseptal access, pericardial access, or a combination of both.

### 6.1. PLAATO Device (ev3 Endovascular, Plymouth, MN, USA)

**Figure 1:**
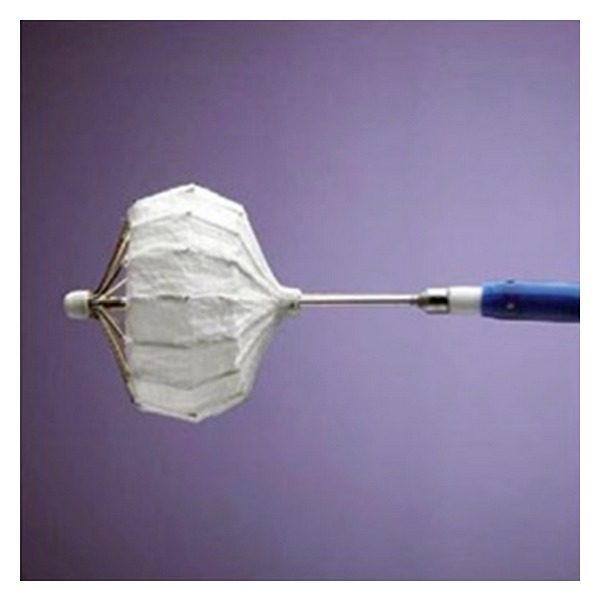
The PLAATO device shown mounted on its delivery catheter. (*Image courtesy of Nature Publishing Group.*)

 In 2001, the percutaneous LAA transcatheter occlusion (PLAATO) system became the first percutaneous LAA exclusion device employed in humans. It consists of a self-expanding nitinol cage covered with a blood-impermeable polytetrafluoroethylene membrane (Figure 1). Anchors along the maximum circumference of the device hold it securely to the LAA wall. The device is deployed in the LAA via transseptal catheterization under fluoroscopic and TEE guidance.

Clinical experience has been reported in three studies. Sievert et al. implanted the device in fifteen patients with chronic AF and contraindications to anticoagulation, achieving 100% procedural success with one incident of hemopericardium during LAA access [28]. An international multicenter registry of 111 patients with contraindications to anticoagulation demonstrated a 97% implant success rate and a 6% adverse event rate, including one death [29]. The ten-month stroke rate of 2.2% compared favorably to the CHADS_2_ predicted rate of 6.3%. Finally, the five-year North American registry reported 100% procedural success in sixty-four AF patients at high risk for stroke [30]. In this cohort the five-year stroke rate was 3.8%, again superior to the expected 6.6% (relative risk reduction of 42%). Despite these promising early experiences, the manufacturer did not pursue further refinement of the device and instead withdrew it from the market in 2006 due to financial reasons.

### 6.2. AMPLATZER Cardiac Plug (St. Jude Medical, Plymouth, MN, USA)

**Figure 2:**
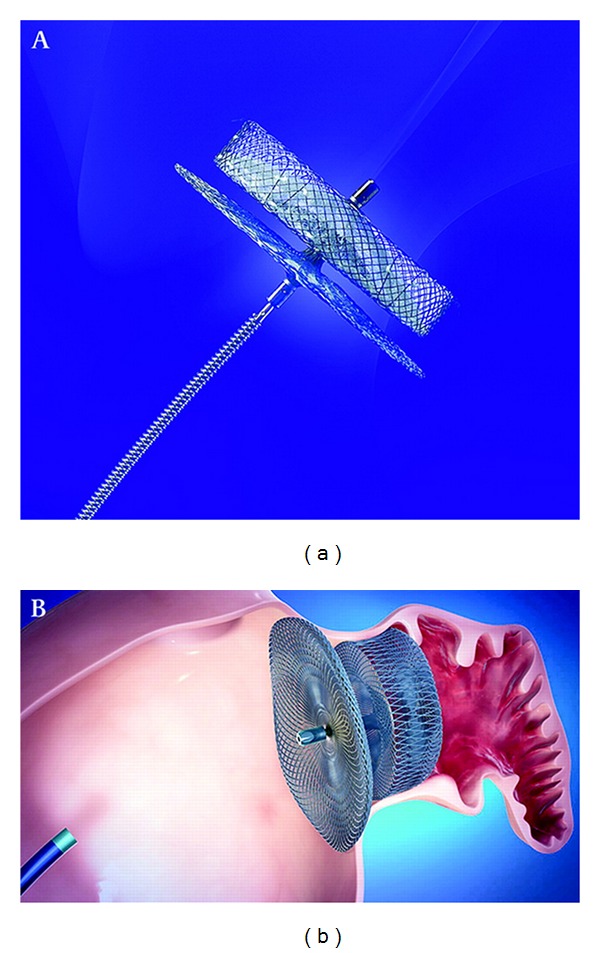
The AMPLATZER Cardiac Plug shown (a) mounted on its delivery catheter and (b) properly deployed at the ostium of the left atrial appendage. (*Image courtesy of BMJ Publishing Group Ltd and British Cardiovascular Society.*)

Following the success of the AMPLATZER Septal Occluder for patent foramen ovale and atrial septal defect closure, the AMPLATZER Cardiac Plug (ACP) was developed specifically for the LAA (Figure 2). The device consists of a self-expanding nitinol mesh constructed as a distal lobe (designed to prevent migration) and a proximal disk (designed to occlude the LAA ostium). The lobe and disk are connected by an articulating waist to accommodate anatomic variation among patients. The device is also delivered to the LAA via transseptal catheterization.

The initial human trials, conducted in Europe, demonstrated a 96% procedural success rate in 137 patients [31]. Serious complications occurred in ten patients (ischemic stroke in three; device embolization in two, both percutaneously recaptured; transient ischemia in two; pericardial effusions in five). The initial Asian-Pacific experience in twenty patients was also recently published, highlighting a 95% procedural success rate and complications in three patients (catheter-related thrombus, coronary artery air embolism, and TEE-induced esophageal injury); one year followup showed no incidence of stroke or death [32]. Importantly, ACP implantation protocols have not involved periprocedural anticoagulation, instead employing dual antiplatelet therapy for one month followed by aspirin monotherapy for six months. The ACP has received CE mark approval and is currently in Phase I clinical trials in the United States (http://clinicaltrials.gov/ NCT01118299).

### 6.3. WATCHMAN LAA Closure Device (Atritech, Plymouth, MN, USA)

**Figure 3:**
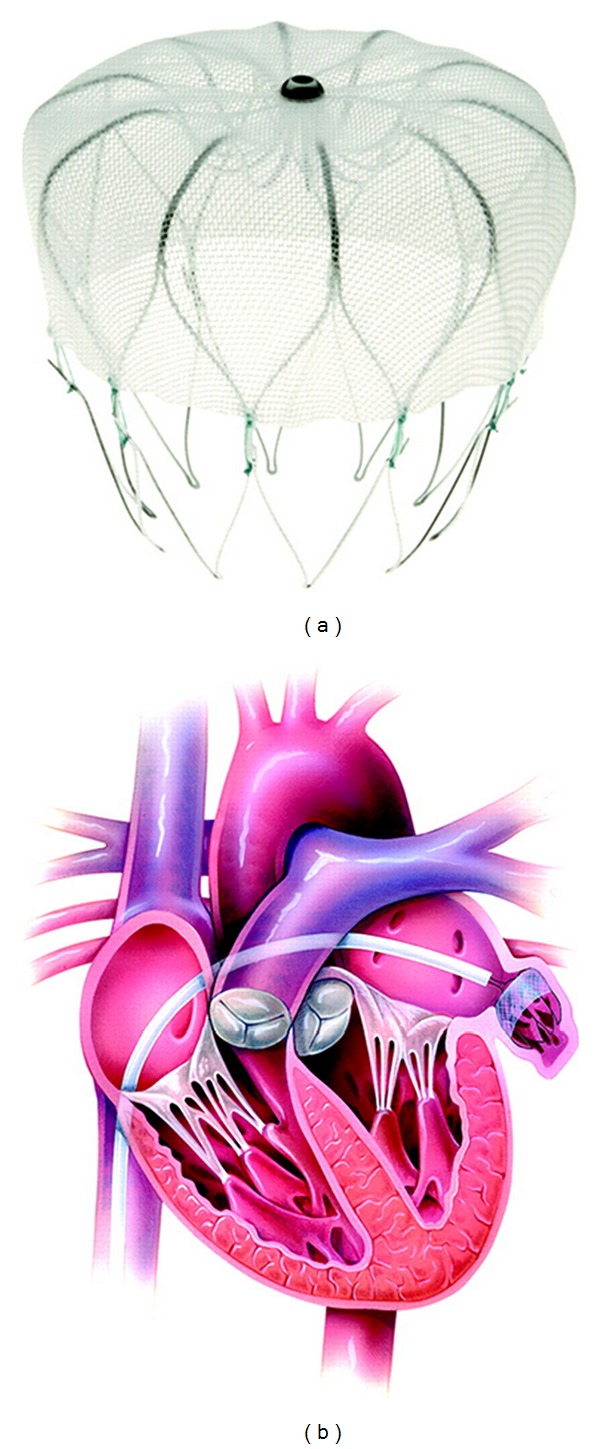
The WATCHMANLAA Closure Device (a) highlighting the permeable membrane covering its left atrial face and (b) properly deployed within the ostium of the left atrial appendage. (*Image courtesy of BMJ Publishing Group Ltd and British Cardiovascular Society.*)

**Figure 4:**
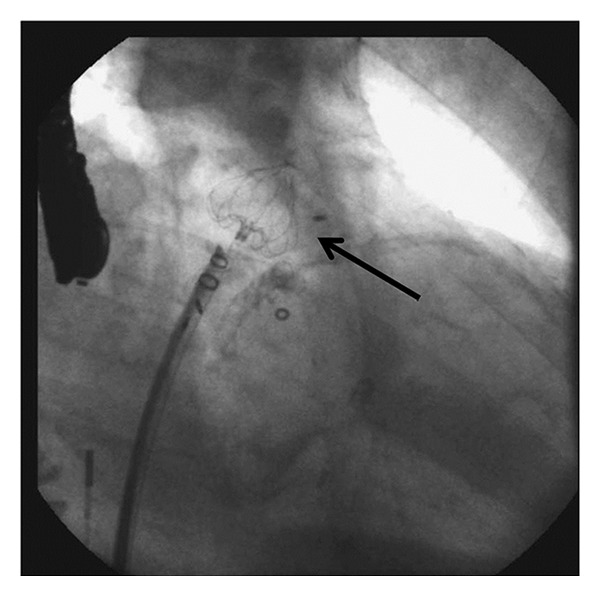
Fluoroscopic image of the WATCHMAN device (arrow) deployed in the left atrial appendage.

 The WATCHMAN device, first implanted in 2002, shares similarities with the ACP and PLAATO systems. It consists of a self-expanding, open-ended nitinol frame with fixation anchors and a polyethylene membrane (Figures 3 and 4). It also has a catheter-based transseptal delivery system. With this device, the membrane is permeable and covers only the part of the device exposed to the left atrium. The WATCHMAN device protocols have required warfarin for at least 6 weeks to prevent thrombus formation prior to endothelialization of the device. Warfarin has been discontinued once a follow-up TEE demonstrates complete occlusion of the LAA. Recently, a strategy of substituting dual antiplatelet therapy for warfarin postimplantation was evaluated in 150 patients in the ASAP registry. Results suggest that this strategy may be safe and effective in patients with contraindications even to short-term warfarin (Heart Rhythm Society Scientific Sessions 2012). 

After the initial feasibility studies, which included a major product redesign, a landmark randomized clinical trial compared the WATCHMAN device with warfarin therapy [33–35]. In PROTECT-AF, 707 patients from fifty-nine centers in the USA and Europe were randomized 2 : 1 to device versus standard warfarin therapy. These patients did not have contraindications to warfarin and their stroke risk was somewhat lower than the PLAATO population (68% had a CHADS_2_ score of 1 or 2). The trial was designed to assess noninferiority of WATCHMAN to standard warfarin therapy. After 1065 patient-years of followup, the primary efficacy endpoint (stroke, systemic embolism, or cardiovascular or unexplained death) was superior in the WATCHMAN group over the warfarin group (3.0% versus 4.9% per 100 patient-years) and achieved the criteria for noninferiority. However, the primary safety endpoint (excessive bleeding or procedure-related complications) was significantly worse in the WATCHMAN group (7.4% versus 4.4% per 100 patient-years). Periprocedural complications included 22 pericardial effusions (4.8%), four air emboli (0.9%), and three device embolizations (0.6%). On the other hand, the warfarin group had a higher incidence of major bleeding (4.1% versus 3.5%) and hemorrhagic stroke (2.5% versus 0.2%). Overall implantation success was 91% and at six months, 92% of patients in the WATCHMAN group were able to discontinue warfarin after a TEE confirmed complete LAA closure.

Of note, procedure-related and device-related adverse events were greater in the first half of PROTECT AF than in the second half, highlighting the learning curve involved with device implantation [36]. The adverse events rate continued to remain low in the Continued Access Protocol (CAP) Registry of 460 patients. A second randomized trial, PREVAIL, is currently underway in hopes of obtaining FDA approval for the WATCHMAN device. This trial is similar in design to PROTECT AF but with stricter inclusion criteria.

**Figure 5:**
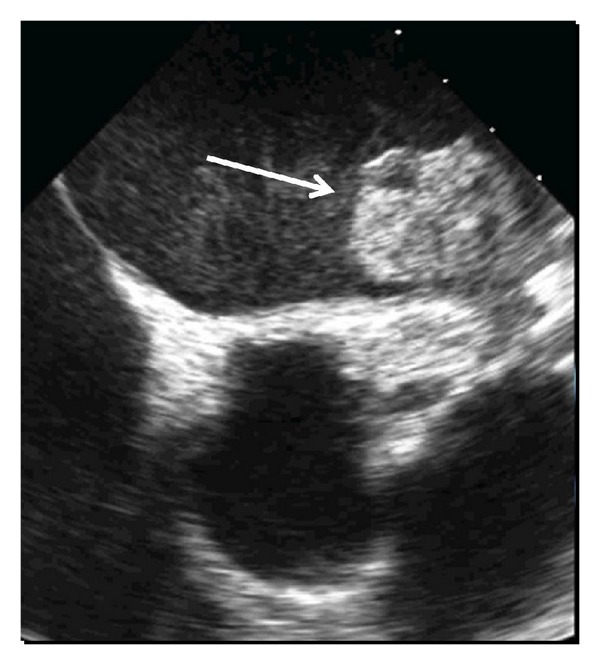
Transesophageal echocardiographic image of a large thrombus (arrow) on a WATCHMAN device several months after anticoagulation was discontinued.

 Thus far, the WATCHMAN device is the only transseptal LAA exclusion device that has demonstrated non-inferiority to warfarin for stroke prevention. However, concerns remain regarding peri-procedural complications and thrombus formation on the device prior to endothelialization (Figure 5).

### 6.4. LARIAT Suture Delivery System (SentreHeart, Palo Alto, CA, USA)

**Figure 6:**
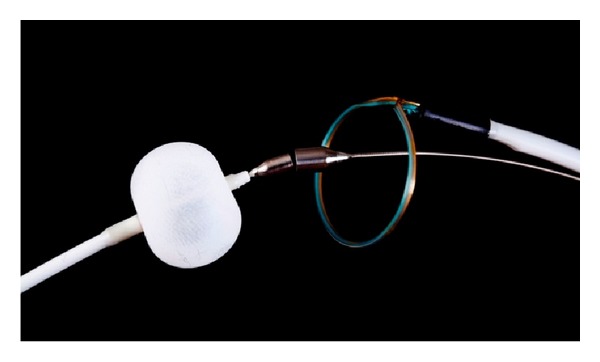
Major components of the LARIAT Suture Delivery System, including magnet-tipped guidewires, endovascular balloon catheter, and epicardial snare mounted with a pretied suture loop. (*Image courtesy of SentreHeart, Inc.*)

**Figure 7:**
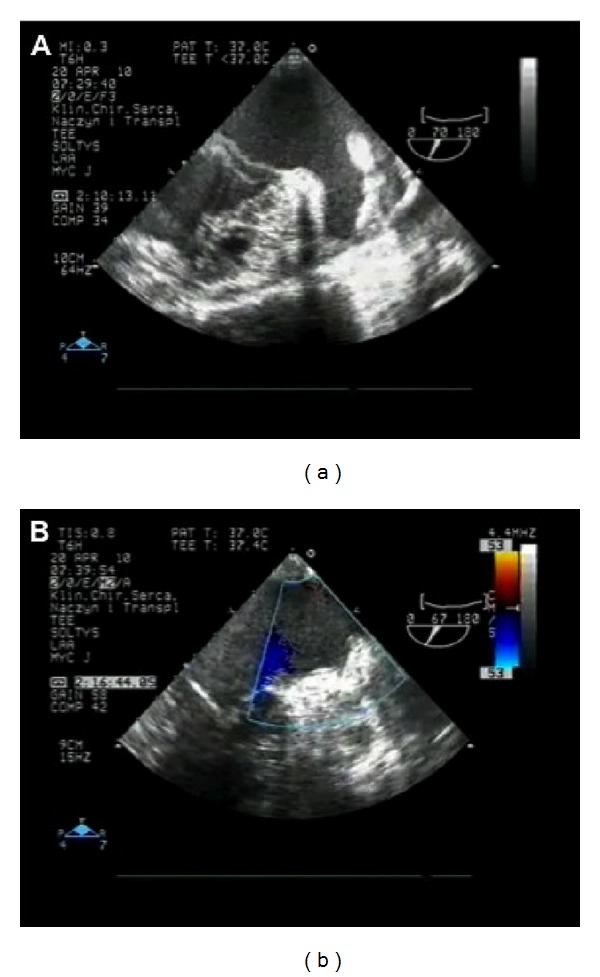
Transesophageal echocardiographic images of the left atrial appendage (a) prior to and (b) immediately after LARIAT suture deployment, highlighting acute LAA exclusion. (*Image courtesy of SentreHeart, Inc.*)

**Figure 8:**
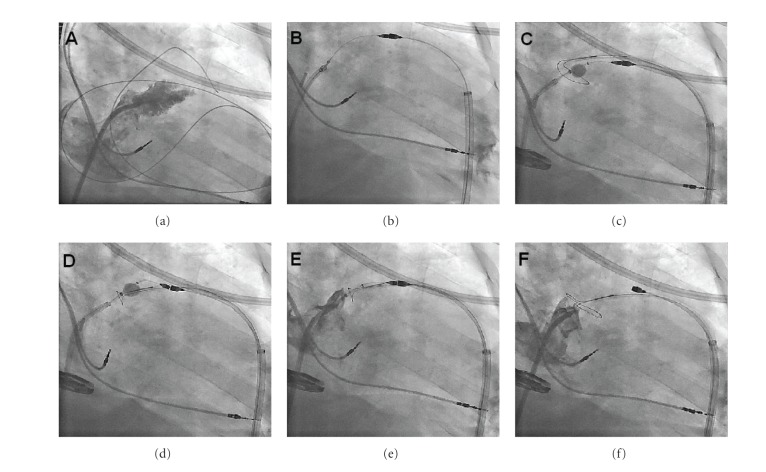
Fluoroscopic sequence of the LARIAT procedure. (a) After transseptal and pericardial access, baseline left atrial angiography identifies the left atrial appendage. (b) Magnet-tipped endocardial and epicardial guidewires make contact across the wall of the left atrial appendage. (c) The balloon catheter is inflated just within the ostium of the left atrial appendage, guiding the placement of the epicardial snare. (d) The snare is tightened at the base of the left atrial appendage. (e) The balloon catheter is pulled back and left atrial angiography confirms occlusion of the left atrial appendage. (f) The suture is cinched down permanently, the snare is retracted, and a final left atrial angiogram reconfirms complete occlusion of the left atrial appendage.

 The newest LAA exclusion device with promising human data is the LARIAT suture delivery system, which received FDA approval in 2009 for soft-tissue occlusion. This hybrid system involves the epicardial and transseptal placement of magnet-tipped guidewires, forming a single rail for the delivery of an endocardial balloon and an epicardial snare with a pretied suture loop (Figure 6). The inflated endocardial balloon acts as a marker for the placement of the epicardial snare around the base of the LAA. Under fluoroscopic and TEE guidance, the suture is then released and cinched to ligate the LAA at its base (Figure 7). Importantly, LAA closure can be evaluated in real time with TEE and left atrial angiography prior to irreversible suture delivery. If closure is not satisfactory, the snare can be opened and repositioned (Figure 8). 

There are several advantages to this approach, including complete control of the pericardial space in the event of cardiac perforation, lack of any endovascular hardware left behind, and possible elimination of the need for postprocedure anticoagulation. The major disadvantage of the LARIAT system is the need for simultaneous transseptal and pericardial access. In addition, anatomic variables can limit candidacy for the device, such as LAA diameter greater than 40 mm, posteriorly rotated LAA, or pericardial adhesions from prior cardiac surgery or pericarditis.

Initial experience in a canine model has confirmed the safety and feasibility of the LARIAT system [37]. In the first human experience, ten patients successfully underwent LAA exclusion with the LARIAT suture, one of whom required thorascopic removal of the snare owing to pectus excavatum [38]. Complete exclusion was confirmed in all ten patients using left atrial angiography and TEE. In a single center, nonrandomized study (PLACE II), LAA exclusion with the LARIAT system was attempted in 89 patients and was successful in 85 (96%). There were three adverse events (3.3%) involving bleeding—two pericardial related, one transseptal related. There were no device-related complications or embolic strokes. Persistent LAA closure (defined as less than 1 mm residual flow) was achieved in 95% of patients at day ninety and 98% of patients at one year (Heart Rhythm Society Scientific Sessions 2012).

## 7. Conclusions

Stroke prevention in AF presents significant challenges as well as opportunities. Current treatment strategies with systemic anticoagulation, while effective, are fraught with limitations involving poor compliance, intolerance, and inconvenience. While newer oral anticoagulants overcome many of these limitations, all anticoagulants suffer from an unavoidable lifelong commitment to medication and elevated bleeding risk.

 Given that the LAA is the source of thromboembolism in the vast majority of patients with AF and stroke, a newer paradigm of targeting the LAA has naturally evolved. Several strategies are available, although surgical LAA removal will likely not have a large role as a stand-alone procedure due to its significant morbidity. The minimally invasive strategies involve foreign body occlusion of the LAA ostium and pericardial suture ligation of the LAA base.

 Several questions remain regarding LAA exclusion. Aside from the PLAATO device, which is no longer available, the information regarding long-term durability of percutaneous LAA exclusion is not yet available. Even after acute procedural success, there is commonly a small diverticulum or “beak” left behind at the LAA ostium. In light of the surgical data that incomplete closure is worse than no closure at all [26], there are concerns about the thrombogenicity of this unnatural diverticulum.

 The data also highlight that success rates are operator and experience dependent. As every new procedure necessarily involves a learning curve, the hope is that the second- and third- generation data with LAA exclusion will show improving procedural success rates with decreasing complication rates. The WATCHMAN experience has already demonstrated this.

 The ultimate dominance of one percutaneous technique over the rest is unlikely. A more likely outcome is that device selection will be tailored to patient characteristics. For instance, prior cardiac surgery or pericardial adhesions would make endocardial occlusion devices more feasible than the LARIAT system. On the other hand, an absolute contraindication to antiplatelet drugs or oral anticoagulation makes the LARIAT system more attractive as it appears to have no requirement for postprocedure anticoagulation. Similarly, a patient deemed at high risk for infection may benefit from the LARIAT system given its lack of endovascular hardware.

An even larger issue is the selection of appropriate candidates for these devices. Current focus has been on patients with intolerance or contraindications to warfarin. Whether these devices will be offered as an equal (or preferred) alternative to anticoagulation remains to be seen. PROTECT AF confirmed the non-inferiority of LAA exclusion to warfarin, but superiority data is still lacking. In addition, all LAA exclusion trials excluded patients with valvular AF or with prosthetic valves; the role of LAA exclusion in these patients is unknown. Finally, to date the comparison arm for these devices has only been warfarin. As some of the newer anticoagulants have reduced bleeding risk compared with warfarin, it is possible that the benefit of mechanical LAA exclusion would diminish in head-to-head trials against the newer agents.

 The ultimate goal of LAA exclusion is to replace the lifelong need for anticoagulation with a single procedure associated with a small upfront risk and tremendous long-term benefit. This paradigm rests on the assumption that thromboembolism in AF is due solely to the anatomic presence of the LAA. However, data suggests that AF is associated with a systemic hypercoagulable state which may contribute to stroke risk in an independent and meaningful way [39]. This argues against discontinuation of anticoagulation, regardless of the patency of the LAA. Larger and longer-term studies will help shed light on this important question.

 Despite the remaining challenges, LAA exclusion represents a promising alternative to systemic anticoagulation for the prevention of stroke in patients with AF. Already, studies have established that LAA exclusion is a viable option in patients with intolerance or contraindications to anticoagulation. Whether LAA exclusion is a superior strategy to anticoagulation in all AF patients remains to be seen. In addition, whether mechanical LAA exclusion reduces risk of stroke over the long term will require further clinical trials.
